# Copeptin in acute decompensation of liver cirrhosis: relationship with acute-on-chronic liver failure and short-term survival

**DOI:** 10.1186/s13054-017-1894-8

**Published:** 2017-12-21

**Authors:** Annarein J. C. Kerbert, Hein W. Verspaget, Àlex Amorós Navarro, Rajiv Jalan, Elsa Solà, Daniel Benten, François Durand, Pere Ginès, Johan J. van der Reijden, Bart van Hoek, Minneke J. Coenraad, for the CANONIC Study Investigators of the EASL-CLIF Consortium

**Affiliations:** 1 Department of Gastroenterology-Hepatology, Leiden University Medical Center, PO Box 9600, Leiden, The Netherlands; 2 Liver Unit/EASL-CLIF Data Center, Hospital Clínic de Barcelona, Barcelona, Spain; 3 Liver Failure Group, UCL Institute for Liver and Digestive Health, UCL Medical School, Royal Free Hospital, London, UK; 4 Liver Unit, Hospital Clínic de Barcelona, University of Barcelona, Barcelona, Spain; 5 Department of Medicine, University Medical Center Hamburg-Eppendorf, Hamburg, Germany; 6 Hepatology and Liver Intensive Care Unit, Hospital Beaujon, Clichy, France

**Keywords:** Acute-on-chronic liver failure, Cirrhosis, Organ failure, Biomarker, Copeptin

## Abstract

**Background:**

Acute-on-chronic liver failure (ACLF) is characterized by the presence of acute decompensation (AD) of cirrhosis, organ failure, and high short-term mortality rates. Hemodynamic dysfunction and activation of endogenous vasoconstrictor systems are thought to contribute to the pathogenesis of ACLF. We explored whether copeptin, a surrogate marker of arginine vasopressin, is a potential marker of outcome in patients admitted for AD or ACLF and whether it might be of additional value to conventional prognostic scoring systems in these patients.

**Methods:**

All 779 patients hospitalized for AD of cirrhosis from the CANONIC database with at least one serum sample available for copeptin measurement were included. Presence of ACLF was defined according to the CLIF-consortium organ failure (CLIF-C OF) score. Serum copeptin was measured in samples collected at days 0–2, 3–7, 8–14, 15–21, and 22–28 when available. Competing-risk regression analysis was applied to evaluate the impact of serum copeptin and laboratory and clinical data on short-term survival.

**Results:**

Serum copeptin concentration was found to be significantly higher in patients with ACLF compared with those without ACLF at days 0–2 (33 (14–64) vs. 11 (4–26) pmol/L; *p* < 0.001). Serum copeptin at admission was shown to be a predictor of mortality independently of MELD and CLIF-C OF scores. Moreover, baseline serum copeptin was found to be predictive of ACLF development within 28 days of follow-up.

**Conclusions:**

ACLF is associated with significantly higher serum copeptin concentrations at hospital admission compared with those with traditional AD. Copeptin is independently associated with short-term survival and ACLF development in patients admitted for AD or ACLF.

**Electronic supplementary material:**

The online version of this article (doi:10.1186/s13054-017-1894-8) contains supplementary material, which is available to authorized users.

## Background

Acute decompensation (AD) of liver cirrhosis is characterized by the occurrence of major complications of the underlying liver disease and is the main cause of hospitalization in cirrhotic patients. Acute-on-chronic liver failure (ACLF) is a life-threatening syndrome that occurs in patients with AD, and is characterized by organ failure and often requires admission to the intensive care unit (ICU) [1, 2]. Several non-evidence-based working definitions have been proposed for ACLF [1, 3, 4]. In order to define clear diagnostic criteria for this syndrome, the European Association for the Study of the Liver—Chronic Liver Failure (EASL-CLIF) consortium has performed the Acute-on-Chronic Liver Failure in Cirrhosis (CANONIC) study [2]. In that study, a large cohort of patients hospitalized for AD were prospectively followed and ACLF was found to be a distinct entity in patients with AD, as it was characterized by the presence of organ failure and a high short-term mortality rate [2]. The activation of endogenous vasoconstrictor systems as an adaptive response to a decreased effective circulating blood volume in cirrhotic patients with a hyperdynamic circulation is thought to be associated with the development of organ failure in ACLF [5, 6]. Conventional prognostic scoring systems in cirrhosis, such as the Model for End-stage Liver disease (MELD) and Child-Pugh score, do not adequately account for risks associated with hemodynamic derangement and organ failure. The recently developed CLIF-consortium organ failure (CLIF-C OF) score has been shown to be superior to the MELD and Child-Pugh score in predicting prognosis in ACLF patients [7]. However, no marker reflecting the degree of activation of endogenous vasoconstrictor systems has been included in this score. Arginine vasopressin (AVP) is a hypothalamic neurohormone which is secreted into the blood stream by the neurohypophysis upon stimuli, such as hyperosmolarity, arterial hypotension, hypovolemia, and physiological stress. Due to its role in both hemodynamic homeostasis and the endogenous stress response, which is also known to be associated with outcome in acute illness, we hypothesized that the AVP system may be of particular prognostic value in critically ill cirrhotic patients. Circulating AVP concentration as such is not suitable due to its instability in serum and poor reproducibility [8]. Copeptin is a stable cleavage product of the C-terminal part of the AVP precursor and is secreted together with AVP in equimolar amounts [9, 10]. Copeptin is therefore generally considered a surrogate marker for AVP. The present study aimed to assess in a large study population of patients admitted for AD or ACLF: 1) copeptin as a prognostic biomarker of short-term survival and disease progression; and 2) whether copeptin might be of additional prognostic value to conventional prognostic scoring systems in cirrhosis and ACLF.

## Methods

### Study population

The present study is an ancillary study of the prospective observational CANONIC study [2] (Additional file 1). Written informed consent was obtained from each patient included in that study and the study protocol conformed to the ethical guidelines of the 1975 Declaration of Helsinki as reflected a priori by the individual institution’s Medical Ethics Committees as described elsewhere [2]. Between February and September 2011, 1349 patients hospitalized for AD of cirrhosis in 29 liver units in eight European countries were included in the CANONIC study. The HCB—IDIBAPS Biobank in Barcelona (Spain) manages the CANONIC database and storage of biomaterials. For 779 patients included in the CANONIC study, a blood sample drawn at hospital admission was available for copeptin measurement and these patients were therefore included in the present ancillary study. ACLF was defined by the CLIF-C OF score [7], adapted from the original chronic liver failure-sequential organ failure assessment (CLIF-SOFA) score and specifically designed for the use in cirrhotic patients with AD [2]. Demographics, clinical characteristics, and laboratory measurements were collected at the time of study enrolment. Patients were followed-up until 28 days after study enrolment. Survival data and events (mortality and liver transplantation) were collected at set time points of 28 days and 3, 6, and 12 months of follow-up.

### Copeptin measurements

Serum samples for copeptin measurements were obtained at days 0–2 (*n* = 779), 3–7 (*n* = 205), 8–14 (*n* = 138), 15–21 (*n* = 12), and 22–28 (*n* = 71) after study enrolment and stored at –80 °C. Serum copeptin measurements were performed in 50 μL plasma samples using an immunoassay in the chemiluminescence-coated tube format (B.R.A.H.M.S., Kryptor, GmbH, Hennigsdorf, Germany). The reference range of serum copeptin in healthy individuals is 1–12 pmol/L with median values of < 5 pmol/L [11, 12].

### Statistical analysis

Discrete variables are shown as counts (percentage) and continuous variables as mean ± standard deviation (SD). Data with a skewed distribution are expressed as median (interquartile range; IQR) and were log-transformed prior to statistical analysis. A *p* value ≤ 0.05 was considered statistically significant.

The relation between ACLF grades and serum copeptin was analyzed using the Kruskal Wallis test and Wilcoxon signed rank test when appropriate. Spearman’s rank order correlation analysis was performed to explore possible correlations between serum copeptin concentration and age, prognostic scoring systems, blood pressure, and laboratory data. In survival analysis, competing-risk regression models according to the method of Fine and Gray [13] were used to assess the prognostic value of copeptin on short-term (28- and 90-day) mortality. In these models, liver transplantation was considered as a competing risk factor in order to adjust for interdependence. Another competing-risk regression model was performed in order to assess the impact of serum copeptin changes over time on survival. To explore the impact of serum copeptin levels on the disease course in patients with ACLF, the definitions ‘improvement’, ‘worsening’, and ‘steady’ ACLF course were applied as previously described by Gustot et al. [14]. Variables with a *p* < 0.05 in univariate regression analyses and age were included in multivariate models. The MELD and CLIF-C OF scores were separately evaluated with copeptin in multivariate models in order to explore whether serum copeptin concentration is associated with outcome independently of these scores. Individual variables included in these scores were not included in multivariate models in order to avoid collinearity. To assess its predictive ability, the Concordance (C-) index of serum copeptin for 28- and 90-day mortality was calculated and its additional predictive value to that of the MELD and CLIF-C OF scores was assessed. A binary logistic regression model was performed in order to identify the independent predictive factors of ACLF development during the complete 28-days of follow-up.

## Results

### Patient characteristics

Baseline demographics and clinical characteristics are shown in Table 1. A comparison between the baseline characteristics of the study cohort of this ancillary study and the whole CANONIC cohort is provided in Additional file 2. At the time of enrolment in the study, 139 (17.8%) patients had ACLF (grade I, *n* = 80; grade II, *n* = 51; grade III, *n* = 8). Serum copeptin at admission was significantly higher in patients with ACLF compared with those without (33 (14–64) vs. 11 (4–26) pmol/L; *p* < 0.001). Significant differences between these patient groups were also found for mean arterial blood pressure (MAP), diastolic blood pressure (DBP), and, as expected, the presence of clinical features such as ascites, hepatic encephalopathy, systemic inflammatory response syndrome (SIRS), sepsis, and bacterial infections. Serum bilirubin, creatinine, and C-reactive protein (CRP) concentrations, white blood cell count (WBC), and international normalized ratio (INR) were all significantly more elevated in the subgroup of patients with ACLF at enrolment compared with those without. Consequently, prognostic scores (Child-Pugh, MELD, CLIF-COF) were significantly higher in ACLF (Table 1).

**Table 1:** Baseline characteristics of 779 cirrhotic patients hospitalized for acute decompensation of cirrhosis

Variable	All patients (*n* = 779)	No ACLF (*n* = 640)	ACLF (*n* = 139)	*p* value^a^
Age (years)	58 ± 12	58 ± 12	58 ± 11	0.893
Gender (male)	512 (65.7)	421 (65.8)	91 (65.5)	0.944
Background				
Diabetes	190 (24.9)	151 (24.0)	39 (29.1)	0.219
Coronary heart disease	37 (5.1)	30 (5.0)	7 (5.5)	0.796
Etiology				
Alcohol	471 (61.0)	374 (58.9)	97 (70.8)	0.010
Hepatitis B	42 (5.7)	37 (6.2)	5 (3.8)	0.282
Hepatitis C	235 (31.9)	197 (32.7)	38 (28.4)	0.333
NAFLD	33 (4.5)	24 (4.0)	9 (6.8)	0.159
Cholestatic	17 (2.3)	15 (2.5)	2 (1.5)	0.505
Cryptogenic	43 (5.8)	40 (6.6)	3 (2.3)	0.052
Other	53 (7.2)	45 (7.5)	8 (6.1)	0.570
Physical examination				
SBP (mmHg)	116 ± 18	117 ± 18	114 ± 21	0.238
DBP (mmHg)	67 ± 11	68 ± 11	63 ± 11	<0.001
MAP (mmHg)	83 ± 12	84 ± 12	80 ± 13	< 0.001
Clinical features				
Ascites	691 (88.7)	557 (87.0)	134 (96.4)	0.002
Bacterial infection	177 (22.8)	134 (21.0)	43 (31.4)	0.009
SIRS	153 (19.6)	115 (18.0)	38 (27.3)	0.012
Sepsis	37 (4.8)	22 (3.5)	15 (11.0)	< 0.001
HE	240 (30.8)	158 (24.7)	82 (59.0)	< 0.001
ACLF grade I	80 (10.3)	–	80 (57.6)	–
ACLF grade II	51 (6.6)	–	51 (36.7)	–
ACLF grade III	8 (1.0)	–	8 (5.7)	–
Organ failure				
Liver	97 (12.5)	45 (7.0)	52 (37.4)	<0.001
Cerebral	39 (5.0)	16 (2.5)	23 (16.6)	< 0.001
Circulation	22 (2.8)	5 (0.8)	17 (12.2)	< 0.001
Respiratory	12 (1.5)	3 (0.5)	9 (6.5)	< 0.001
Renal	82 (10.5)	0 (0.0)	82 (59.0)	< 0.001
Coagulation	43 (5.5)	15 (2.3)	28 (20.1)	< 0.001
Laboratory data				
Copeptin (pmol/L)	13 (5–32)	11 (4–26)	33 (14–64)	< 0.001
WBC (×10^9^/L)	5.9 (4.1–9.3)	5.7 (4.0–8.3)	8.9 (5.3–13.1)	< 0.001
CRP (mg/L)	18 (7–41)	16 (6–37)	26 (12–50)	< 0.001
Bilirubin (mg/dL)	2.9 (1.5–6.5)	2.8 (1.5–5.5)	6.1 (2.0–16.4)	< 0.001
INR	1.5 (1.3–1.8)	1.4 (1.3–1.7)	1.7 (1.4–2.2)	< 0.001
Creatinine (mg/dL)	0.9 (0.7–1.4)	0.9 (0.7–1.2)	2.2 (1.0–3.1)	< 0.001
Sodium (mmol/L)	135 ± 6	136 ± 5	134 ± 7	0.002
Scores				
Child-Pugh	9.4 ± 2.1	9.2 ± 1.9	10.6 ± 2.2	< 0.001
MELD	18 ± 7	16 ± 5	26 ± 7	< 0.001
CLIF-C OF	7.5 ± 1.7	7.0 ± 1.1	9.9 ± 1.9	< 0.001
Treatments^b^				
ICU admission	102 (13.2)	75 (11.8)	27 (19.4)	0.016
Antibiotics	142 (18.8)	111 (17.9)	31 (23.0)	0.170
Transfusion^c^	87 (11.5)	67 (10.7)	20 (14.8)	0.175
Vasoactive agents^d^	39 (5.1)	21 (3.4)	18 (13.3)	< 0.001
Mechanical ventilation	14 (1.8)	9 (1.4)	5 (3.6)	0.078
Renal replacement therapy	3 (0.4)	1 (0.2)	2 (1.4)	0.027

Variables are expressed as mean ± SD, median (interquartile range), or *n* (%) as appropriate

^a^Comparisons between patients with and without ACLF

^b^At any time during follow-up

^c^Includes transfusion of red cells package, fresh-frozen plasma, platelets, and cryoprecipitates.

^d^Includes any vasoactive drug used for circulatory support, variceal bleeding, or hepatorenal syndrome

*ACLF* acute-on-chronic liver failure, *CLIF-C OF* chronic liver failure-consortium organ failure, *CRP* C-reactive protein, *DBP* diastolic blood pressure, *HE* hepatic encephalopathy, *ICU* intensive care unit, *INR* international normalized ratio, *MAP* mean arterial blood pressure, *MELD* Model for End-stage Liver Disease, *NAFLD* non-alcoholic fatty liver disease, *SBP* systolic blood pressure, *SIRS* systemic inflammatory response syndrome, *WBC* white blood cell count

### Baseline serum copeptin and association with kidney and circulatory function and ACLF

**Fig. 1:**
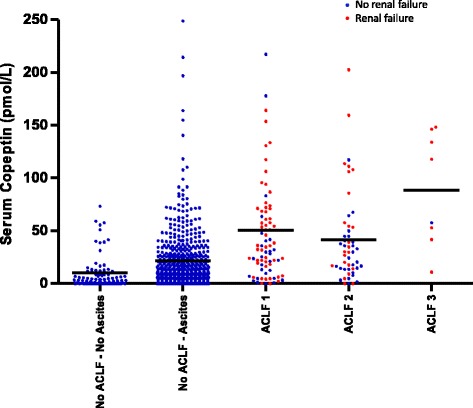
Association of ACLF grades with serum copeptin concentration and the presence of renal failure. Distribution of serum copeptin concentration within subgroups of patients with acute-on-chronic liver failure (*ACLF*) and patients with and without ascites and no ACLF at time of admission for acute decompensation of cirrhosis. Dots represent serum copeptin concentrations of individual patients. Horizontal lines denote median values

Patients with ACLF grade III at enrolment displayed the highest serum copeptin concentration at hospital admission. However, median serum copeptin concentrations did not consistently increase along with the grade of ACLF (grade I, 32 (15–66) pmol/L; grade II, 30 (14–53) pmol/L; grade III, 88 (47–140) pmol/L). Serum copeptin concentration did not significantly differ between grade I and II (*p* = 0.460), but was significantly higher in grade III compared with grade II (*p* = 0.029) and grade III compared with grade I + II (*p* < 0.001). Figure 1 shows the association of ACLF grades with serum copeptin concentration and the presence of renal failure (*n* = 82). In ACLF grade I, serum copeptin was significantly higher in patients with renal failure (as defined by the CLIF-C OF score [7]) at hospital admission compared with those without (49 (21–72) vs. 23 (6–38) pmol/L; *p* = 0.014). However, no significant difference in serum copeptin was found between patients with and without renal failure in ACLF grade II (35 (17–106) vs. 22 (13–40) pmol/L; *p* = 0.132). In ACLF grade III, seven out of eight patients had renal failure (118 (42–146) pmol/L; Fig. 1). Of all 82 patients with renal failure at admission, 47 (57.3%) patients recovered from renal failure during follow-up (i.e., return of serum creatinine to < 2 mg/dL). Baseline serum copeptin concentration was significantly lower in those who recovered from renal failure during follow-up than in those who did not (35 (14–69) vs. 59 (28–114) pmol/L; *p* = 0.019).

When comparing patients with and without circulatory failure (i.e., the use of vasopressors for circulatory support at hospital admission according to the CLIF-COF score; *n* = 22), serum copeptin levels were found to be significantly higher in the circulatory failure group (42 (11–64) vs. 13 (5–31) pmol/L; *p* = 0.002). In addition, a weak but statistically significant inverse correlation between serum copeptin and MAP and DBP at enrolment was found. Significant correlations with serum copeptin were also found for serum sodium and serum creatinine concentration, WBC, INR, prothrombin time, and Child-Pugh, MELD, and CLIF-C OF score (Table 2). Besides the use of vasopressors, the use of beta blockers may also potentially impact on serum copeptin concentrations. However, there was no significant difference in median serum copeptin concentration between patients who did and did not receive beta-blocker therapy (9.9 (5.0–27.3) vs. 13.4 (4.7–31.8) pmol/L; *p* = 0.375).

**Table 2:** Associations of clinical parameters and prognostic scoring systems with serum copeptin concentration

Variable	Correlation coefficient with serum copeptin (*r*)	*p* value
Age	0.188	< 0.001
Scores		
Child-Pugh	0.213	< 0.001
MELD	0.276	< 0.001
CLIF-C OF	0.203	< 0.001
Laboratory data		
WBC^a^	0.228	< 0.001
Bilirubin^a^	0.064	0.075
Prothrombin time^a^	0.216	< 0.001
INR^a^	0.100	0.006
Creatinine^a^	0.408	< 0.001
Sodium	–0.104	0.004
Physical examination		
SBP	–0.055	0.124
DBP	–0.080	0.027
MAP	–0.072	0.047

^a^Variable was log-transformed prior to statistical analysis

*CLIF-C OF* chronic liver failure-consortium organ failure, *DBP* diastolic blood pressure, *INR* international normalized ratio, *MAP* mean arterial blood pressure, *MELD* Model for End-stage Liver Disease, *SBP* systolic blood pressure, *WBC* white blood cell count

### Serum copeptin measurements during follow-up and relation with clinical outcome

Sequential serum samples for copeptin measurement were available for 421 out of 779 patients included in the study; 179 patients had a sample available at both days 0–2 and 3–7, and 85 patients had samples available at days 0–2, 3–7, and 8–14. Overall serum copeptin concentration decreased in the first week of follow-up (Additional file 3). Delta serum copeptin in the first week after hospital admission (i.e., serum copeptin at days 3–7 minus serum copeptin at days 0–2) was –3 (–29 to 9) pmol/L. Median serum copeptin at days 3–7 was found to be significantly higher in ACLF patients with a worsening or steady disease course (*n* = 48) during the follow-up period of 28 days compared with patients with improvement of the ACLF course (*n* = 52; 43 (21–70) vs. 22 (10–36) pmol/L; *p* = 0.003) [14]. In contrast, median serum copeptin at days 0–2 and delta copeptin did not significantly differ between these groups (Additional file 4). However, in the whole study population, median serum copeptin at days 0–2 was significantly more elevated in ACLF patients with a worsening or steady disease course (*n* = 68) compared with those showing improvement of the disease (*n* = 71) during follow-up (41 (18–91) vs. 30 (13–53) pmol/L; *p* = 0.030). No correlations were found with delta copeptin and delta values of markers of renal and liver function, blood pressure, inflammation, and MELD and CLIF-C OF scores (Additional file 5).

Delta copeptin in the first week after hospital admission was found not to be associated with survival in the subgroup of 179 patients with serum samples available at both days 0–2 and 3–7.

### Survival analysis

At 28 days after enrolment in the study, 63 (8.1%) patients had died and 24 (3.1%) had received a liver transplantation. After 90 days of follow-up, 132 (16.9%) patients had died and 63 (8.1%) were transplanted. Serum copeptin was consistently found to be significantly higher in patients who died or were transplanted as compared to those who were still alive without liver transplantation at 28 and 90 days follow-up (28 days, 39 (18–68) vs. 12 (4–28) pmol/L, *p* < 0.001; 90 days, 27 (11–56) vs. 11 (4–26) pmol/L, *p* < 0.001).

Results of univariate competing risk survival analysis of clinical and laboratory data in relation to 28- and 90-day mortality are shown in Additional files 6 and 7, respectively. A strong association was found for serum copeptin concentration at days 0–2 and survival at both time points.

In multivariate analysis, copeptin together with WBC and MELD or CLIF-C OF score was found to independently predict 28-day mortality (Table 3). Moreover, C-indices of the MELD and CLIF-C OF score for predicting 28-day mortality improved significantly by incorporating serum copeptin (*p* = 0.004 and *p* = 0.037, respectively; Table 4).

**Table 3:** Independent predictive factors of 28-day and 90-day mortality in 779 patients admitted for acute decompensation of cirrhosis; multivariate analysis

	HR (95% CI)	*p* value
Mortality at 28 days		
Model 1: MELD score		
MELD score	1.10 (1.06–1.14)	< 0.001
Copeptin^a^	1.55 (1.20–2.01)	< 0.001
WBC^a^	1.82 (1.23–2.95)	0.014
Model 2: CLIF-C OF score		
CLIF-C OF score	1.43 (1.25–1.64)	< 0.001
Copeptin^a^	1.53 (1.19–1.97)	0.001
WBC^a^	1.92 (1.18–3.13)	0.009
Mortality at 90 days		
Model 1: MELD score		
MELD score	1.11 (1.07–1.15)	< 0.001
Copeptin^a^	1.15 (0.97–1.37)	0.113
Age	1.03 (1.01–1.05)	0.006
WBC^a^	1.77 (1.26–2.47)	< 0.001
Sodium	0.96 (0.93–0.99)	0.041
Model 2: CLIF-C OF score		
CLIF-C OF score	1.39 (1.23–1.56)	< 0.001
Copeptin^a^	1.22 (1.02–1.45)	0.032
Age	1.02 (1.00–1.04)	0.032
WBC^a^	1.87 (1.32–2.65)	< 0.001
Sodium	0.95 (0.92–0.98)	0.002

^a^Variable was log-transformed prior to statistical analysis

*CI* confidence interval, *CLIF-C OF* chronic liver failure-consortium organ failure, *HR* hazard ratio, *MELD* Model for End-stage Liver Disease, *WBC* white blood cell count

**Table 4:** C-indices of copeptin in association with MELD score and CLIF-C OF score at 28 days and 90 days of follow-up

	C-index (95% CI)	*p* value
Mortality at 28 days		
Copeptin^a^	0.723 (0.660–0.787)	
MELD	0.766 (0.707–0.826)	Reference
MELD + copeptin^a^	0.796 (0.742–0.849)	0.004
CLIF-C OF	0.739 (0.668–0.809)	Reference
CLIF-C OF + copeptin^a^	0.798 (0.748–0.849)	0.037
Mortality at 90 days		
Copeptin^a^	0.654 (0.606–0.702)	
MELD	0.749 (0.707–0.792)	Reference
MELD + copeptin^a^	0.757 (0.716–0.798)	0.160
CLIF-C OF	0.699 (0.651–0.746)	Reference
CLIF-C OF + copeptin^a^	0.728 (0.686–0.771)	0.077

^a^Variable was log-transformed prior to statistical analysis

*CLIF-C OF* chronic liver failure-consortium organ failure, *CI* confidence interval, *MELD* Model for End-stage Liver Disease

At 90-days of follow-up, copeptin was found to be an independent predictive factor for mortality as well, together with the CLIF-C OF score, age, WBC, and serum sodium concentration (Table 3, model 2). However, in the MELD score model, copeptin was found not to be an independent predictive factor for mortality at this time point (Table 3, model 1). At 90 days of follow-up, C-indices of the MELD and CLIF-C OF score for predicting mortality did not significantly improve by incorporating serum copeptin (*p* = 0.160 and *p* = 0.077, respectively; Table 4).

**Fig. 2:**
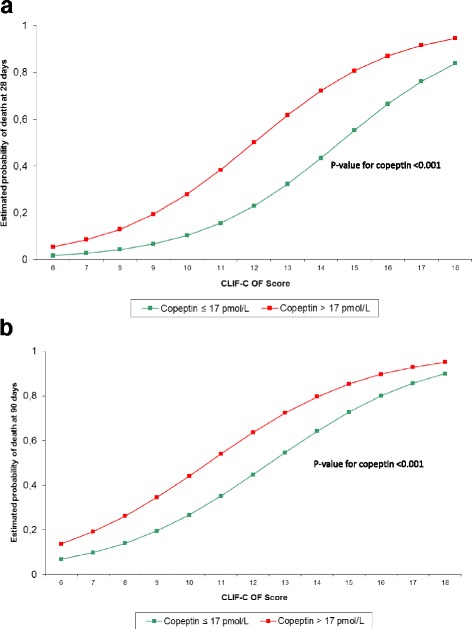
Association of the estimated probability of death using the chronic liver failure-consortium organ failure (*CLIF-C OF*) score at 28 days (**a**) and 90 days (**b**) of follow-up, stratified according to serum copeptin concentration. The optimal cut-off point of serum copeptin in predicting 28- and 90-day mortality was defined using the Youden Index

Figure 2 shows the estimated probability of death after 28 and 90 days of follow-up using the CLIF-C OF score and stratification according to serum copeptin concentration, showing that high serum copeptin concentrations have an additional negative impact on mortality risk. The optimal cut-off point of serum copeptin in predicting mortality at 28 and 90 days used in Fig. 2 was calculated using the Youden Index.

Finally, a multivariate binary logistic regression model was performed in order to identify the independent predictive factors for ACLF development within 28 days of follow-up (*n* = 71). Serum copeptin, together with WBC and INR, was found to be an independent predictive factor for ACLF development (odds ratio (OR) 1.40, 95% confidence interval (CI) 1.09–1.80; *p* = 0.009) (Additional file 8). With the use of the Youden Index we defined an optimal cut-off point of serum copeptin of 13.6 pmol/L in predicting ACLF development during follow-up. In multivariate analysis, serum copeptin > 13.6 pmol/L remained a predictor of ACLF development, independently of WBC and INR (OR 2.94, 95% CI 1.67–5.16; *p* < 0.001) (Additional file 8).

## Discussion

In this study, we assessed the prognostic ability of copeptin, a surrogate marker of AVP, in patients admitted for AD of cirrhosis or ACLF. The results demonstrate that the presence of ACLF is accompanied by significantly higher serum copeptin levels at admission compared with those with traditional AD. In addition, serum copeptin was found to be independently associated with short-term outcome and was shown to provide additional prognostic information to the MELD and CLIF-C OF scores.

The ideal prognostic biomarker for predicting short-term ACLF development and mortality in patients with AD is one that is elevated at the time of onset of AD, is involved in the pathophysiology of disease progression, and can therefore help in directing and monitoring therapy. Markers reflecting hemodynamic systemic changes in cirrhotic patients, such as the hepatic venous pressure gradient (HVPG) and MAP, are well known to be associated with the presence of organ failure and prognosis in cirrhosis [15–21]. In clinical practice, a prognostic biomarker reflecting the degree of circulatory derangement may therefore be of importance since it may help to distinguish between patients who are at a higher risk of developing organ failure and short-term mortality. It may also add prognostic information to conventional prognostic scoring systems in cirrhosis, such as the MELD and Child-Pugh score, which take into account indirect, nonspecific, or subjective markers of hemodynamic derangement such as ascites and creatinine concentration. Recent studies have shown an association of high serum copeptin levels with hemodynamics, such as portal hypertension (HVPG > 12 mmHg) [20] and a decreased cardiac output [21]. In this study, the role of copeptin in hemodynamic homeostasis was shown by the finding of a weak, but significant inverse correlation between MAP and DBP with copeptin. The weakness of this association may be explained by the fact that, besides peripheral vasodilation, copeptin levels may also be influenced by a number of other stimuli, such as hyperosmolarity, physiological and psychological stress, and medication (i.e., diuretics, beta blockers and vasopressors) [22].

To date, the prognostic value of copeptin in the setting of liver cirrhosis has been investigated in a few studies [23–26]. The results of these studies show that serum copeptin levels increase along with the severity of liver disease, as defined by the Child-Pugh class [23, 25]. Moreover, circulating copeptin concentration was found to predict short- and long-term transplant-free mortality in patients with various stages of cirrhosis [23–26]. In addition, a prospectively conducted study showed the ability of plasma copeptin to predict the development of cirrhosis-related complications and death within 3 months after hospitalization [26]. However, no data have been reported on the prognostic value of serum copeptin in an unselected cohort of patients admitted for AD and ACLF. Currently, several scoring systems are in use for risk stratification in critically ill cirrhotic patients, such as the MELD and Child-Pugh score. The CLIF-C OF score was recently developed as a simplification of the CLIF-SOFA score to diagnose and grade ACLF [7]. Its prognostic accuracy was found to be significantly higher than that of the MELD and Child-Pugh scores in patients with AD or ACLF [7]. In the current study, it was shown that serum copeptin predicts the risk for short-term mortality, independently of the CLIF-C OF (28- and 90-day mortality) and MELD (28-day mortality) scores. Moreover, incorporation of serum copeptin in the MELD and CLIF-C OF scores improved their prognostic ability for 28-day mortality. Serum copeptin measured at days 0–2 and 3–7 after hospital admission was found to be associated with the course of ACLF during short-term follow-up. Finally, serum copeptin at days 0–2 after hospital admission was found to independently predict short-term ACLF development. On the other hand, no association between delta serum copeptin over time and disease course and survival was found. This finding requires further assessment in larger prospectively conducted trials in which serum copeptin can be obtained in all patients at set and well-defined time points.

Deterioration of systemic hemodynamic function is traditionally thought to play a key role in the development of multi-organ failure in ACLF [2, 3, 5]. However, in the light of new knowledge, it is now thought that the presence of systemic inflammation in cirrhosis is the key event in ACLF development [5]. This ‘systemic inflammation hypothesis’ proposes that ACLF develops as a result of aggravation of systemic inflammation and associated systemic circulatory dysfunction which is already present in AD. This hypothesis was tested in a recent study by Clarìa et al. [27]. The authors found that AD is associated with very high plasma levels of cytokines and oxidized albumin and that ACLF develops when there is a further increase in these inflammatory mediators. In addition, Clarìa et al. found that markers of systemic circulatory dysfunction (i.e., copeptin and renin) were significantly more elevated in patients with ACLF compared with those without. Remarkably, in contrast to markers of systemic inflammation, these hemodynamic biomarkers did not consistently increase through ACLF grade I–III, which is consistent with our findings. This finding suggests that hemodynamic dysfunction is present in ACLF, but is not directly associated with the severity of ACLF. This implicates a role for pathophysiological mechanisms other than circulatory dysfunction, such as systemic inflammation, contributing to the severity of ACLF. Nevertheless, in the current study, copeptin was found to be a strong and independent prognostic factor for short-term outcome, especially at 28 days of follow-up. Besides reflecting the activity of the AVP system due to the systemic hemodynamic changes, the prognostic ability of copeptin may also be explained by its non-specificity. As mentioned previously, copeptin may be influenced by various stimuli, such as hyperosmolarity and physiological and psychological stress [22]. In the setting of acute hospitalization for AD or ACLF, this non-specificity may be its strength. As the complexity of the pathogenesis of ACLF is high, single, organ-specific biomarkers may oversimplify the pathology of the disease. Copeptin has been shown to be a reliable novel marker of endogenous stress levels, mirroring moderate stress levels more subtly than cortisol [28]. The prognostic ability of copeptin, as a marker of an acute and generalized hemodynamic stress response, has extensively been studied in general populations of patients admitted to the emergency department and ICU, showing promising results [29, 30]. For future studies, it might be interesting to explore the prognostic ability of copeptin specifically in cirrhotic patients with AD or ACLF admitted to the ICU.

Some limitations concerning the present study have to be considered. Firstly, plasma copeptin levels were markedly higher in patients with ACLF and renal failure than in ACLF patients without renal failure. This may indicate that elevated plasma copeptin levels may not only reflect an increased release of AVP by the neurohypophysis, but also a decreased clearance rate of copeptin in patients with ACLF and renal failure. Although copeptin is thought to be, at least partly, excreted by the kidneys [9, 31], it is currently not entirely clear how copeptin is removed from the body. Future studies should focus on the potential causal relationship between renal function and serum copeptin levels and whether this impacts on the prognostic ability of copeptin in these patients. Secondly, the effect of possible confounding factors such as the use of certain drugs was not (sufficiently) studied due to the lack of information on use (diuretics), moment of blood sampling and drug administration (vasopressors), specification of the indication (vasopressors), and dose (vasopressors and beta blockers). Thirdly, consecutive copeptin measurements were only performed in a limited number of patients. To further explore copeptin as a prognostic marker in AD and ACLF, and the prognostic ability of copeptin in predicting ACLF development, a prospectively conducted and larger cohort study in which copeptin measurements are sequentially performed would be interesting. Finally, another potential confounding factor is the presence of (cirrhotic) cardiomyopathy in this population, especially because of the relatively large proportion of patients with alcoholic liver disease (61.0%). Copeptin has been found to be associated with the presence of both acute and chronic heart failure and is associated with prognosis [32]. Therefore, it would be relevant to take into account cardiac function in future studies.

## Conclusions

Serum copeptin levels are significantly more increased in patients with ACLF compared with those with traditional AD. Moreover, serum copeptin is a predictor of mortality in cirrhotic patients admitted for AD, independently of MELD and CLIF-COF scores. Serum copeptin shows the potential to add relevant prognostic information to these prognostic scoring systems. Altogether, these findings suggest that serum copeptin is an interesting potential prognostic marker in hospitalized cirrhotic patients with AD and ACLF.

## Additional files

Additional file 1: Table S1. List of CANONIC study investigators in alphabetical order. (PDF 123 kb)

Additional file 2: Table S2. Comparison of baseline characteristics between the complete cohort of the CANONIC study (*n* = 1349) and the cohort of the current ancillary study (*n* = 779). (PDF 38 kb)

Additional file 3: Table S3. Consecutive serum copeptin concentrations. (PDF 26 kb)

Additional file 4: Table S4. Changes in laboratory values over time according to the clinical course of ACLF in patients with ACLF at baseline and a sample available at days 0–2 and days 3–7 (*n* = 100). (PDF 31 kb)

Additional file 5: Table S5. Correlation coefficients of changes in laboratory and clinical values of patients with a sample available at days 0–2 and days 3–7 (*n* = 179). (PDF 23 kb)

Additional file 6: Table S6. Parameters associated with 28-day survival in a population of 779 patients admitted for acute decompensation of cirrhosis. Univariate analysis. (PDF 26 kb)

Additional file 7: Table S7. Parameters associated with 90-day survival in a population of 779 patients admitted for acute decompensation of cirrhosis. Univariate analysis. (PDF 26 kb)

Additional file 8: Table S8. Independent predictive factors of ACLF development in 600 patients admitted for acute decompensation of cirrhosis and without ACLF. Multivariate analysis including copeptin as a continuous variable (A) and using its optimal cut-off point in predicting ACLF (B). (PDF 19 kb)

## Abbreviations

ACLF: Acute-on-chronic liver failure
AD: Acute decompensation
AVP: Arginine vasopressin
CANONIC: Acute-on-Chronic Liver Failure in Cirrhosis
CI: Confidence interval
CLIF-C OF: chronic liver failure-consortium organ failure
CLIF-SOFA: chronic liver failure-sequential organ failure assessment
CRP: C-reactive protein
DBP: Diastolic blood pressure
EASL-CLIF: European Association for the Study of the Liver—Chronic Liver Failure
HVPG: Hepatic venous pressure gradient
ICU: Intensive care unit
INR: International normalized ratio
IQR: Interquartile range
MAP: Mean arterial blood pressure
MELD: Model for End-stage Liver Disease
OR: Odds ratio
SD: Standard deviation
SIRS: Systemic inflammatory response syndrome
WBC: White blood cell count
