# Supplementary material for: Copeptin in acute decompensation of liver cirrhosis: relationship with acute-on-chronic liver failure and short-term survival
Source: Crit Care. 2017 Dec 21;21:321. doi: 10.1186/s13054-017-1894-8 (PMC5740749; doi:10.1186/s13054-017-1894-8)
Supplement: Supplementary file 1 — List of CANONIC study investigators in alphabetical order. (PDF 123 kb) [file 13054_2017_1894_MOESM1_ESM.pdf]

Supplementary table 1. List of CANONIC Study Investigators in alphabetical order

1. Patricia Aguilar Melero, Hospital Universitario Reina Sofía. Centro de Investigación Biomédica en Red Enfermedades Hepáticas y Digestivas (CIBERehd). Instituto Maimónides de Investigación Biomédica de Córdoba.(IMIBIC) Córdoba, Spain.

2. Rafael Bañares , Hospital General Universitario Gregorio Marañón, Instituto de Investigación

Sanitaria Gregorio Marañón (IiSGM), Centro de Investigación Biomédica en Red Enfermedades

Hepáticas y Digestivas (CIBERehd), School of Medicine. Universidad Complutense. Madrid Spain.

3. Massimo Bocci, Department of Gastroenterology and Hepato-Pancreatology, Erasme Hospital, Université Libre de Bruxelles, Brussels, Belgium.

4. María-Vega Catalina, Hospital General Universitario Gregorio Marañón, Instituto de Investigación Sanitaria Gregorio Marañón (IiSGM), Centro de Investigación Biomédica en Red Enfermedades Hepáticas y Digestivas (CIBERehd), Madrid Spain.

5. Jun Liong Chin, Liver Unit, St Vincent's University Hospital, Dublin, Ireland.

6. Minneke J. Coenraad, Leiden University Medical Centre, Department of Gastroenterology-

Hepatology, Leiden. The Netherlands.

7. Audrey Coilly, Centre Hépato-Biliaire, Hôpital Paul-Brousse, Assistance Publique-Hôpitaux de Paris, Villejuif, France.

8. Livia Dorn, Department of Gastroenterology and Hepatology, Innsbruck Medical

University,Innsbruck, Austria

9. Angelo Gatta, Department of Medicine, University of Padova, Padova, Italy.

10. Ludmila Gerber, Department of Medicine I, JW Goethe University Hospital, Frankfurt,Germany

11. Henning Grøenbæk, Department of Medicine V, Unit of hepatology and gastroenterology,Aarhus University Hospital, Aarhus, Denmark

12. Isabel Graupera, Liver Unit, Hospital Clinic, University of Barcelona, Centro de Investigación Biomédica en Red Enfermedades Hepáticas y Digestivas

(CIBERehd), Barcelona, Spain.

13. Monica Guevara, Liver Unit, Hospital Clinic, University of Barcelona, Centro de Investigación Biomédica en Red Enfermedades Hepáticas y Digestivas

(CIBERehd), Barcelona.Spain

14. AnneKristin Hausen; Department of Internal Medicine I, University Hospital of Bonn, Bonn,

Germany.

15. Stine Karlsen, Department of Medicine V, Unit of Hepatology and Gastroenterology, Aarhus

University Hospital, Aarhus, Denmark

16. Ansgar W. Lohse Department of Gastroenterology and Hepatology, University Medical Center Hamburg-Eppendorf, Hamburg, Germany.

17. Caterina Maggioli, Semeiotica Medica, Policlinico S. Orsola-Malpighi Department of Medical and Surgical Sciences University of Bologna, Bologna, Italy.

18. Daniel Markwardt, Liver Center Munich, Department of Medicine 2, Klinikum der LMU München- Grosshadern, Munich, Germany.

19. Javier Martinez, Servicio de Gastroenterología, Hospital Universitario Ramón y Cajal, Madrid, Spain.

20. Alfredo Marzano; Division of Gastroenterology and Hepatology, San Giovanni Battista Hospital. University of Turin. Turin. Italy.

21. Manuel de la Mata García Hospital Universitario Reina Sofía. Centro de Investigación Biomédica en Red Enfermedades Hepáticas y Digestivas (CIBERehd). Instituto Maimónides de Investigación Biomédica de Córdoba.(IMIBIC) Córdoba, Spain.

22. Francisco Mesonero, Servicio de Gastroenterología, Hospital Universitario Ramón y Cajal, Madrid, Spain.

23. Rajeshwar P Mookerjee, Institute of Liver and Digestive Health, Liver Failure Group, Royal Free Campus, London, United Kingdom.

24. Christophe Moreno, Department of Gastroenterology and Hepato-Pancreatology, Erasme Hospital, Université Libre de Bruxelles, Brussels, Belgium

25. Bernhard Morrell; University Clinic of Visceral Surgery and Medicine of Berne. Berne, Switzerland.

26. Christian Mortensen Department of Gastroenterology, Hvidovre Hospital, University of

Copenhagen. Copenhagen,Denmark

27. Frederik Nevens, Department of Liver and Biliopancreatic Diseases, University Hospital

Gasthuisberg, KU Leuven, Leuven.Belgium

28. Markus Peck-Radosavljevic, Department of Gastroenterology and Hepatology, Medical University of Vienna, Vienna, Austria.

29. Mario Rizzetto; Division of Gastroenterology and Hepatology, San Giovanni Battista Hospital. University of Turin. Turin. Italy.

30. Antonietta Romano, Department of Medicine, University of Padova, Padova, Italy.

31. Didier Samuel, Centre Hépato-Biliaire, Hôpital Paul Brousse, Assistance Publique-Hôpitaux de Paris, Villejuif, France.

32. Tilman Sauerbruch, Department of Internal Medicine I, University Hospital of Bonn, Bonn

Germany.

33. Macarena Simon-Talero. Servicio de Hepatologia, Hospital Vall d'Hebron, Universitat Autònoma de Barcelona, Centro de Investigación Biomédica en Red Enfermedades Hepáticas y Digestivas (CIBERehd), Barcelona.Spain.

34. Elsa Solà, Liver Unit, Hospital Clinic, University of Barcelona, Centro de Investigación Biomédica en Red Enfermedades Hepáticas y Digestivas

(CIBERehd), Barcelona, Spain.

35. German Soriano, Department of Gastroenterology, Hospital de la Santa Creu i Sant Pau,

Barcelona, Universitat Autònoma de Barcelona, Centro de Investigación Biomédica en Red

Enfermedades Hepáticas y Digestivas (CIBERehd), Instituto de Salud Carlos III, Barcelona, Spain

36. Jan Sperl, Department of Hepatogastroenterology, Institute for Clinical and Experimental Medicine, Prague, Czech Republic.

37. Walter Spindelboeck Division of Gastroenterology and Hepatology, Department of Internal

Medicine, Medical University of Graz, Graz, Austria

38. Christian Steib, Liver Center Munich, Department of Medicine 2, Klinikum der LMU München-Grosshadern, Munich, Germany.

39. Dominique Valla, Service d'Hépatologie, Hôpital Beaujon, Assistance Publique Hôpitaux de Paris, Clichy; Inserm U773, Centre de Recherche Biomédicale Bichat-Beaujon CRB3, Clichy and Paris; and Université Paris Diderot-Paris 7, Paris, France.

40. Len Verbeke, Department of Liver and Biliopancreatic Diseases, University Hospital Gasthuisberg, KU Leuven, Leuven.Belgium

41. Hans Van Vlierberghe, Department of Gastroenterology and Hepatology, Ghent University

Hospital, Ghent, Belgium.

42. Henninge Wege, Department of Gastroenterology and Hepatology, University Medical Center Hamburg-Eppendorf, Hamburg, Germany.

43. Chris Willars, Liver Intensive Care Unit, King's College Hospital, London, United Kingdom.

44. Maria Yago Baenas, Institute of Liver and Digestive Health, Liver Failure Group, Royal Free

Campus,London, United Kindgom.

45. Giacomo Zaccherini, Semeiotica Medica, Policlinico S. Orsola-Malpighi Department of Medical and Surgical Sciences University of Bologna, Bologna, Italy.
