# Supplementary material for: Copeptin in acute decompensation of liver cirrhosis: relationship with acute-on-chronic liver failure and short-term survival
Source: Crit Care. 2017 Dec 21;21:321. doi: 10.1186/s13054-017-1894-8 (PMC5740749; doi:10.1186/s13054-017-1894-8)
Supplement: Supplementary file 2 — Comparison of baseline characteristics between the complete cohort of the CANONIC study (n = 1349) and the cohort of the current ancillary study (n = 779). (PDF 38 kb) [file 13054_2017_1894_MOESM2_ESM.pdf]

Supplementary table 2. Comparison of baseline characteristics between the complete cohort of the CANONIC study (n=1349) and the cohort of the current ancillary study (n=779).

Variable	All CANONIC patients (n=1349)	Ancillary study (n=779)	p-value
**Age (years)**	57 ± 12	58 ± 12	0.335
**Gender (male), n (%)**	854 (63.3)	512 (65.7)	0.262
**Background, n (%)**			
Diabetes	302 (22.8)	190 (24.9)	0.259
Coronary heart disease	71 (5.5)	37 (5.1)	0.657
**Etiology, n (%)**			
Alcohol	820 (61.3)	471 (61.0)	0.901
Hepatitis B	77 (6.0)	42 (5.7)	0.824
Hepatitis C	374 (28.9)	235 (31.9)	0.162
NAFLD	73 (5.7)	33 (4.5)	0.253
Cholestatic	35 (2.7)	17 (2.3)	0.573
Cryptogenic	75 (5.8)	43 (5.8)	0.998
Other	105 (8.2)	53 (7.2)	0.444
**Physical exam**			
SBP (mmHg)	116 ± 19	116 ± 18	0.997
DBP (mmHg)	67 ± 12	67 ± 11	0.893
MAP (mmHg)	83 ± 12	83 ± 12	0.947
**Clinical features, n (%)**			
Ascites	1209 (89.6)	691 (88.7)	0.509
Bacterial infection	325 (24.2)	177 (22.8)	0.490
SIRS	305 (7.3)	153 (69.9)	0.332
Sepsis	73 (5.5)	37 (4.8)	0.504
HE	460 (34.1)	240 (30.8)	0.117
**Laboratory data**			
WBC (x 10^9/L)	6.2 (4.3-9.2)	5.9 (4.1-9.3)	0.313
CRP (mg/L)	18 (7-43)	18 (7-41)	0.330
Bilirubin (mg/dL)	3.0 (1.6-7.4)	2.9 (1.5-6.5)	0.196
Prothrombine time (s)	18 (15-24)	18 (16-25)	0.303
INR	1.5 (1.3-1.9)	1.5 (1.3-1.8)	0.119
Creatinine (mg/dL)	0.9 (0.7-1.4)	0.9 (0.7-1.4)	0.695
Sodium (mmol/L)	135 ± 6	135 ± 6	0.421
**Scores**			
Child-Pugh	9.7 ± 2.1	9.4 ± 2.1	0.003
MELD	19 ± 7	18 ± 7	0.024
CLIF-C OF	7.8 ± 2.1	7.5 ± 1.7	0.002
**Treatments, n (%)**			
ICU admission	198 (14.8)	102 (13.2)	0.314
Antibiotics	255 (19.7)	142 (18.8)	0.633
Transfusion[$]	152 (11.7)	87 (11.5)	0.872
Variceal bleeding*	143 (11.0)	87 (11.5)	0.733
Vasoactive agents[#]	83 (6.4)	39 (5.1)	0.250
Mechanical ventilation	42 (3.1)	14 (1.8)	0.068
Renal replacement therapy	21 (1.6)	3 (0.4)	0.014
Beta-blockers use	133 (10.3)	66 (8.7)	0.259

ACLF, acute-on-chronic liver failure; NAFLD: non-alcoholic fatty liver disease; PBC: primary biliary cholangitis; SBP, systolic blood pressure, DBP, diastolic blood pressure; MAP, mean arterial blood pressure; SIRS, systemic inflammatory response syndrome; HE: hepatic encephalopathy; WBC: white blood cell count; CRP: C-reactive protein; INR: international normalized ratio; MELD: Model for End-stage Liver Disease; MELD-Na: Model for End-Stage Liver Disease with incorporation of serum sodium concentration; CLIF-C OF: CLIF-Consortium Organ Failure Score

Variables are expressed as mean ± SD, median (IQR) or numbers and percentage.

[a] p-value of comparisons between patients with and without ACLF
* at any time during follow-up
[$] includes transfusion of red cells package, fresh-frozen plasma, platelets, and cryoprecipitates.
[#]includes any vasoactive drug used for circulatory support, variceal bleeding or hepatorenal syndrome.
