# Supplementary material for: Copeptin in acute decompensation of liver cirrhosis: relationship with acute-on-chronic liver failure and short-term survival
Source: Crit Care. 2017 Dec 21;21:321. doi: 10.1186/s13054-017-1894-8 (PMC5740749; doi:10.1186/s13054-017-1894-8)
Supplement: Supplementary file 3 — Consecutive serum copeptin concentrations. (PDF 26 kb) [file 13054_2017_1894_MOESM3_ESM.pdf]

Supplementary table 3. Consecutive serum copeptin concentrations.

	Serum copeptin (pmol/L)			
**Group***	**Day 0-2**	**Day 3-7**	**Day 8-14**	**Day 22-28**
**Patients with a sample at day 0-2 (n=779)**	13.3 (4.7-31.9)	n.a.	n.a.	n.a.
**Patients with a sample at day 0-2 and 3-7 (n=179)**	26.6 (7.8-53.4)	21.0 (9.0-41.8)	n.a.	n.a.
**Patients with a sample at day 0-2 till 8-14 (n=85)**	30.3 (10.9-62.7)	24.9 (12.1-49.9)	23.6 (14.3-43.8)	n.a.
**Patients with a sample at day 0-2 till 8-14 and 22-28 (n=38)**	31.3 (5.9-62.7)	23.9 (12.1-49.9)	22.5 (17.1-43.8)	27.3 (12.9-70.6)

n.a., not applicable

*Only 12 patients had a sample available at 15-21 days after hospital admission (data not shown).

Serum copeptin concentration are expressed in pmol/L as median and interquartile range.
