# Supplementary material for: Copeptin in acute decompensation of liver cirrhosis: relationship with acute-on-chronic liver failure and short-term survival
Source: Crit Care. 2017 Dec 21;21:321. doi: 10.1186/s13054-017-1894-8 (PMC5740749; doi:10.1186/s13054-017-1894-8)
Supplement: Supplementary file 4 — Changes in laboratory values over time according to the clinical course of ACLF in patients with ACLF at baseline and a sample available at days 0–2 and days 3–7 (n = 100). (PDF 31 kb) [file 13054_2017_1894_MOESM4_ESM.pdf]

Supplementary table 4. Changes in laboratory values over time according to the clinical course of ACLF in patients with ACLF at baseline and a sample available at day 0-2 and day 3-7 (n=100).

Variable	All patients (n=100)	Patients with improvement of ACLF course (n=52)	Patients with steady or worsening ACLF course (n=48)	p-value*
**Copeptin day 0-2**	36 (17-64)	32 (14-58)	41 (21-86)	0.152
**Copeptin day 3-7**	29 (11-55)	22 (10-36)	43 (21-70)	0.003
**Delta copeptin**	-3 (-29-(9))	-4 (-31-(5))	4 (-29-(21))	0.137
**Creatinine day 0-2**	2.2 (1.1-3.2)	2.0 (0.9-2.7)	2.6 (1.7-3.7)	0.007
**Creatinine day 3-7**	1.4 (1.0-2.2)	1.2 (0.8-1.5)	1.7 (1.3-3.6)	<0.001
**Delta creatinine**	-0.3 (-1.2-(0.0))	-0.4 (-1.1-(0.0))	-0.3 (-1.3-(0.1))	0.712
**Sodium day 0-2**	135 (130-138)	135 (131-139)	134 (127-138)	0.209
**Sodium day 3-7**	136 (133-141)	137 (133-141)	136 (133-141)	0.814
**Delta sodium**	2 (-2-(8))	1 (-3-(7))	4 (0-9)	0.083
**CRP day 0-2**	28 (13-51)	32 (16-55)	20 (7-45)	0.108
**CRP day 3-7**	22 (11-39)	20 (11-36)	25 (11-49)	0.373
**Delta CRP**	-3 (-17-(3))	-7 (-31-(11))	0.2 (-6.6-(13.7))	0.005
**Leucocytes day 0-2**	9.0 (5.2-13.2)	9.1 (4.8-12.8)	8.9 (5.2-13.5)	0.566
**Leucocytes day 3-7**	7.4 (4.3-12.2)	7.3 (3.9-11.7)	8.3 (5.8-14.2)	0.146
**Delta leucocytes**	-0.4 (-2.9-(1.8))	-0.5 (-3.5(1.1))	-0.1 (-2.7-(2.6))	0.341
**Bilirubin day 0-2**	6.1 (2.0-16.0)	3.9 (1.7-12.5)	9.9 (2.5-19.0)	0.038
**Bilirubin day 3-7**	6.3 (2.5-16.9)	4.7 (2.1-10.4)	13 (3-20)	0.028
**Delta bilirubin**	0.2 (-1.0-(1.7))	0.2 (-1.1-(1.6))	0.2 (-1.0-(2.0))	0.743
**INR day 0-2**	1.7 (1.4-2.2)	1.6 (1.3-2.1)	1.8 (1.4-2.4)	0.087
**INR day 3-7**	1.8 (1.4-2.3)	1.6 (1.3-1.9)	1.9 (1.5-2.8)	0.010
**Delta INR**	0.0 (-0.2-(0.2))	0.0 (-0.2-(0.1))	0.1 (-0.1-(0.3))	0.034
**MAP day 0-2**	80 (72-89)	81 (74-89)	79 (67-89)	0.608
**MAP day 3-7**	81 (74-87)	83 (75-86)	80 (73-90)	0.706
**Delta MAP**	0 (-8-(8))	-0.8 (-7.5-(8.3))	3 (-11-(10))	0.633
**SBP day 0-2**	115 (100-130)	115 (104-129)	114 (95-130)	0481
**SBP day 3-7**	112 (104-125)	110 (105-122)	115 (102-129)	0.787
**Delta SBP**	0 (-15-(11))	-4 (-15-(9))	5 (-16-(15))	0.254
**DBP day 0-2**	63 (52-70)	62 (52-70)	63 (54-70)	0.969
**DBP day 3-7**	61 (60-70)	61 (60-70)	62 (55-70)	0.899
**Delta DBP**	0 (-7-(10))	0 (-5-(0))	0 (-10-(10))	0.855
**CLIF-C OF day 0-2**	10 (9-11)	10 (9-11)	10 (9-12)	0.467
**CLIF-C OF day 3-7**	10 (8-11))	8 (7-10)	11 (9-13)	<0.001
**Delta CLIF-OF**	-1 (-1-(0))	-1 (-2-(-1))	0 (-1-(3))	<0.001
**MELD day 0-2**	26 (22-31)	23 (19-28)	29 (25-34)	<0.001
**MELD day 3-7**	25 (20-31)	21 (17-25)	29 (26-36)	<0.001
**Delta MELD**	-1 (-4-(1))	-2 (-6-(0))	1 (-2-(3))	0.002

Delta, value at day 3-7 minus day 0-2; ACLF, acute-on-chronic liver failure; CRP, C-reactive protein; INR, international normalized ratio; MAP, mean arterial blood pressur; SBP, systolic blood pressure; DBP, diastolic blood pressure; CLIF-C OF, CLIF-Consortium organ failure score; MELD, model for end-stage liver disease.
