# Supplementary material for: Copeptin in acute decompensation of liver cirrhosis: relationship with acute-on-chronic liver failure and short-term survival
Source: Crit Care. 2017 Dec 21;21:321. doi: 10.1186/s13054-017-1894-8 (PMC5740749; doi:10.1186/s13054-017-1894-8)
Supplement: Supplementary file 5 — Correlation coefficients of changes in laboratory and clinical values of patients with a sample available at days 0–2 and days 3–7 (n = 179). (PDF 23 kb) [file 13054_2017_1894_MOESM5_ESM.pdf]

Supplementary table 5. Correlation coefficients of changes in laboratory and clinical values of patients with a sample available at day 0-2 and day 3-7 (n=179)

	Correlation coefficient with delta copeptin (r)	p-value
**Delta creatinine**	0.103	0.170
**Delta sodium**	0.131	0.082
**Delta CRP**	0.052	0.553
**Delta leucocytes**	0.046	0.546
**Delta bilirubin**	-0.063	0.401
**Delta INR**	0.021	0.784
**Delta MAP**	-0.034	0.656
**Delta SBP**	-0.096	0.202
**Delta DBP**	0.020	0.793
**Delta CLIF-C OF score**	0.022	0.801
**Delta MELD score**	0.058	0.440

Delta, value at day 3-7 minus day 0-2; CRP, C-reactive protein; INR, international normalized ratio; MAP, mean arterial blood pressur; SBP, systolic blood pressure; DBP, diastolic blood pressure; CLIF-C OF, CLIF-Consortium organ failure score; MELD, model for end-stage liver disease.
