# Supplementary material for: Copeptin in acute decompensation of liver cirrhosis: relationship with acute-on-chronic liver failure and short-term survival
Source: Crit Care. 2017 Dec 21;21:321. doi: 10.1186/s13054-017-1894-8 (PMC5740749; doi:10.1186/s13054-017-1894-8)
Supplement: Supplementary file 6 — Parameters associated with 28-day survival in a population of 779 patients admitted for acute decompensation of cirrhosis. Univariate analysis. (PDF 26 kb) [file 13054_2017_1894_MOESM6_ESM.pdf]

Supplementary table 6. Parameters associated with 28-day survival in a population of 779 patients admitted for acute decompensation of cirrhosis. Univariate analysis.

Variable	Mortality at 28 days		
	Survivors (n=692)	Dead (n=63)	p-value
**Age (years)**	58±12	59±9	0.157
**Gender (male), n (%)**	452 (65.3)	39 (61.9)	0.505
**Physical exam**			
SBP (mmHg)	116±18	118±19	0.324
DBP (mmHg)	67±11	65±10	0.235
MAP (mmHg)	83±12	82±12	0.538
**Clinical features, n (%)**			
Ascites	606 (87.6)	62 (98.4)	0.011
Bacterial infection	152 (22.1)	20 (31.8)	0.067
SIRS	124 (17.9)	24 (38.1)	<0.001
Sepsis	28 (4.1)	7 (11.1)	0.010
HE	197 (28.5)	33 (52.4)	<0.001
**Organ failures, n (%)**			
Liver	66 (9.5)	24 (38.1)	<0.001
Cerebral	27 (3.9)	10 (15.9)	<0.001
Circulatory	14 (2.0)	6 (9.5)	<0.001
Respiratory	9 (1.3)	3 (4.8)	0.033
Renal	62 (9.0)	18 (28.6)	<0.001
Coagulation	31 (4.5)	9 (14.3)	0.001
**Laboratory data**			
Copeptin (pmol/L)	12 (4-28)	39 (18-68)	<0.001
WBC (x 10^9/L)	5.7 (4.0-8.8)	9.0 (6.3-13.3)	<0.001
CRP (mg/L)	16 (6-38)	32 (18-58)	0.016
Bilirubin (mg/dL)	2.7 (1.5-5.7)	6.2 (2.7-19.7)	<0.001
Prothrombine time (s)	18 (15-24)	22 (17-26)	0.026
INR	1.4 (1.3-1.7)	1.8 (1.5-2.3)	<0.001
Creatinine (mg/dL)	0.9 (0.7-1.3)	1.3 (0.8-2.5)	<0.001
Sodium (mmol/L)	135±5	133±8	0.001
**Scores**			
Child–Pugh	9.2±2.0	11.2±1.9	<0.001
MELD	17±6	25±8	<0.001
CLIF-C OF	7±1	9±3	<0.001

SBP, systolic blood pressure, DBP, diastolic blood pressure; MAP, mean arterial blood pressure; SIRS, systemic inflammatory response syndrome; HE: hepatic encephalopathy; WBC: white blood cell count; CRP: C-reactive protein; INR: international normalized ratio; MELD: Model for End-stage Liver Disease; CLIF-C OF: CLIF-Consortium Organ Failure Score.

Variables are expressed as mean ± SD, median (IQR) or numbers and percentage.

24 transplanted patients were considered as a secondary event.
