# Supplementary material for: Copeptin in acute decompensation of liver cirrhosis: relationship with acute-on-chronic liver failure and short-term survival
Source: Crit Care. 2017 Dec 21;21:321. doi: 10.1186/s13054-017-1894-8 (PMC5740749; doi:10.1186/s13054-017-1894-8)
Supplement: Supplementary file 7 — Parameters associated with 90-day survival in a population of 779 patients admitted for acute decompensation of cirrhosis. Univariate analysis. (PDF 26 kb) [file 13054_2017_1894_MOESM7_ESM.pdf]

Supplementary table 7. Parameters associated with 90-day survival in a population of 779 patients admitted for acute decompensation of cirrhosis. Univariate analysis.

Variable	Mortality at 90 days		
	Survivors (n=584)	Dead (n=132)	p-value
**Age (years)**	58±12	59±11	0.177
**Gender (male), n (%)**	385 (65.9)	79 (59.9)	0.117
**Physical exam**			
SBP (mmHg)	116±18	117±19	0.522
DBP (mmHg)	67±11	67±11	0.627
MAP (mmHg)	84±12	83±13	0.794
**Clinical features, n (%)**			
Ascites	502 (86.0)	129 (97.7)	<0.001
Bacterial infection	125 (21.5)	36 (27.5)	0.144
SIRS	108 (18.5)	35 (26.5)	0.018
Sepsis	23 (4.0)	9 (6.9)	0.167
HE	160 (27.4)	60 (45.5)	<0.001
**Organ failures, n (%)**			
Liver	43 (7.4)	40 (30.3)	<0.001
Cerebral	22 (3.8)	14 (10.6)	<0.001
Circulatory	12 (2.1)	7 (5.3)	0.035
Respiratory	9 (1.5)	3 (2.3)	0.370
Renal	42 (7.2)	32 (24.2)	<0.001
Coagulation	22 (3.8)	15 (11.4)	<0.001
**Laboratory data**			
Copeptin (pmol/L)	11 (4-26)	27 (11-56)	<0.001
WBC (x 10^9/L)	5.6 (3.9-8.3)	8.3 (5.8-12.6)	<0.001
CRP (mg/L)	15 (6-35)	35 (18-59)	<0.001
Bilirubin (mg/dL)	2.5 (1.4-5.0)	5.9 (2.6-14.3)	<0.001
Prothrombine time (s)	18 (15-24)	20 (17-27)	0.027
INR	1.4 (1.3-1.7)	1.7 (1.5-2.2)	<0.001
Creatinine (mg/dL)	0.9 (0.7-1.2)	1.2 (0.8-1.9)	<0.001
Sodium (mmol/L)	136±5	132±7	<0.001
**Scores**			
Child–Pugh	9.0±2.0	10.8±1.8	<0.001
MELD	16±6	24±7	<0.001
CLIF-C OF	7±1	9±2	<0.001

SBP, systolic blood pressure, DBP, diastolic blood pressure; MAP, mean arterial blood pressure; SIRS, systemic inflammatory response syndrome; HE: hepatic encephalopathy; WBC: white blood cell count; CRP: C-reactive protein; INR: international normalized ratio; MELD: Model for End-stage Liver Disease; CLIF-C OF: CLIF-Consortium Organ Failure Score

Variables are expressed as mean ± SD, median (IQR) or numbers and percentage.

63 transplanted patients were considered as a secondary event.
