# Supplementary material for: Copeptin in acute decompensation of liver cirrhosis: relationship with acute-on-chronic liver failure and short-term survival
Source: Crit Care. 2017 Dec 21;21:321. doi: 10.1186/s13054-017-1894-8 (PMC5740749; doi:10.1186/s13054-017-1894-8)
Supplement: Supplementary file 8 — Independent predictive factors of ACLF development in 600 patients admitted for acute decompensation of cirrhosis and without ACLF. Multivariate analysis including copeptin as a continuous variable (A) and using its optimal cut-off point in predicting ACLF (B). (PDF 19 kb) [file 13054_2017_1894_MOESM8_ESM.pdf]

Supplementary table 8. Independent predictive factors of ACLF development in 640 patients admitted for acute decompensation of cirrhosis and without ACLF. Multivariate analysis including copeptin as a continuous variable (A) and using its optimal cut-off point of 13.6 pmol/L in predicting ACLF (B).

A.

**Variable**	**ACLF development**	
	**HR (95% CI)**	**p-value**
**Copeptin***	1.40 (1.09-1.80)	0.009
**WBC***	1.83 (1.13-2.94)	0.013
**INR***	16.05 (5.61-45.93)	<0.001

B.

**Variable**	**ACLF development**	
	**HR (95% CI)**	**p-value**
**Copeptin >13.6 pmol/L**	1.40 (1.09-1.80)	0.009
**WBC***	1.85 (1.16-2.96)	0.010
**INR***	15.11 (5.33-42.84)	<0.001

WBC, white blood cell count; INR, international normalized ratio

*Variable was log-transformed prior to statistical analysis
